# Cytoreductive Surgery and Hyperthermic Intraperitoneal Chemotherapy for Gastric Cancer with Synchronous Peritoneal Metastases: Multicenter Study of ‘Italian Peritoneal Surface Malignancies Oncoteam—S.I.C.O.’

**DOI:** 10.1245/s10434-021-10157-0

**Published:** 2021-05-31

**Authors:** Luigi Marano, Daniele Marrelli, Paolo Sammartino, Daniele Biacchi, Luigina Graziosi, Elisabetta Marino, Federico Coccolini, Paola Fugazzola, Mario Valle, Orietta Federici, Dario Baratti, Marcello Deraco, Andrea Di Giorgio, Antonio Macrì, Enrico Maria Pasqual, Massimo Framarini, Marco Vaira, Franco Roviello, the Italian Peritoneal Surface Malignancies Oncoteam (S.I.C.O.)

**Affiliations:** 1 Department of Medicine, Surgery and Neurosciences, Unit of General Surgery and Surgical Oncology, University of Siena, Siena, Italy; 2 Cytoreductive Surgery and HIPEC Unit, Department of Surgery “Pietro Valdoni”, Sapienza University of Rome, Rome, Italy; 3 General and Emergency Surgery, University of Perugia, Perugia, Italy; 4 General, Emergency and Trauma Surgery Department, Bufalini Hospital, Cesena, Italy; 5 Department of Digestive Surgery, IRCCS Regina Elena National Cancer Institute, Rome, Italy; 6 Fondazione IRCCS Istituto Nazionale Dei Tumori di Milano, Peritoneal Surface Malignancies Unit, Milan, Italy; 7 Surgical Unit of Peritoneum and Retroperitoneum, Fondazione Policlinico Universitario A. Gemelli IRCCS, Rome, Italy; 8 Peritoneal Surface Malignancy and Soft Tissue Sarcoma Program, Messina University Medical School Hospital, Messina, Italy; 9 Department of Medical Area, University of Udine, Santa Maria della Misericordia University Hospital Udine, Udine, Italy; 10 Department of Surgery, Morgagni-Pierantoni Hospital, Forlì, Italy; 11 Candiolo Cancer Institute, Unit of Surgical Oncology, FPO-IRCCS, Candiolo, Italy; 12 General, Emergency and Trauma Surgery Department, Pisa University Hospital, Pisa, Italy

## Abstract

**Background:**

The development of multimodality treatment, including cytoreductive surgery (CRS) with heated intraperitoneal chemotherapy (HIPEC), has led to promising results in selected patients with peritoneal disease of gastric origin. The aim of this study was to investigate the short- and long-term outcomes of CRS/HIPEC in the treatment of synchronous peritoneal metastasis in gastric cancer.

**Methods:**

The Italian Peritoneal Surface Malignancies Oncoteam—S.I.C.O. retrospective registry included patients with synchronous peritoneal malignancy from gastric cancer submitted to gastrectomy with CRS and HIPEC between 2005 and 2018 from 11 high-volume, specialized centers.

**Results:**

A total of 91 patients with a median age of 58 years (range 26–75) were enrolled. The median overall survival (OS) time for the whole group of patients was 20.2 months (95% confidence interval [CI] 11.8–28.5] and the median recurrence-free survival (RFS) was 7.3 months (95% CI 4–10.6). The completeness of cytoreduction score (CCS) of 0 and Peritoneal Cancer Index (PCI) score of ≤ 6 groups showed a significantly better long-term survival (median OS 40.7 and 44.3 months, respectively) compared with the incomplete resected groups (median OS 10.7 months, *p* = 0.003) and PCI score of > 6 group (median OS 13.4 months, *p* = 0.005). A significant difference was observed in the survival rate according to neoadjuvant treatment (untreated patients: 10.7 months, 95% CI 5.1–16.2; treated patients: 35.3 months, 95% CI 2.8–67.8; *p* = 0.022).

**Conclusions:**

In referral centers, CRS and HIPEC after neoadjuvant treatment significantly improved survival in selected patients. Patients with a PCI score ≤ 6, complete cytoreduction, negative nodal involvements, and negative cytology had encouraging results, showing a clinically meaningful survival.

The 5-year survival of gastric cancer (GC) patients with advanced or metastatic disease is dramatically poor, accounting for < 10% of patients.^1^ The main drawback for curative resection is the peculiar propensity for peritoneal spreading, found in approximately 30% of patients at the time of primary diagnosis.^2^–^4^ The development of a multimodality treatment strategy, including cytoreductive surgery (CRS) combined with heated intraperitoneal chemotherapy (HIPEC), has recently led to promising results in selected patients with peritoneal disease of gastric origin, thus changing the role of peritoneal disease as a marker for death.^5^ Since its first description by Sugarbaker et al.^6^ and Yonemura et al.^7^ in the 1990s, CRS/HIPEC has progressively shown higher feasibility and efficacy from an oncological point of view. In recent years, similar encouraging results have been obtained by several other research groups,^8^–^10^ particularly in selected groups of GC patients with oligometastatic peritoneal disease. However, although comprehensive treatment, consisting of CRS combined with HIPEC, seems to be the only strategy to improve the long-term survival of selected GC patients with synchronous PM, there is currently a lack of evidence regarding its clinical value.^11^–^13^ Recently, several national registries, such as the BIG-RENAPE in France, the Spanish Group of Peritoneal Oncologic Surgery (GECOP), the German HIPEC register, and other national registries, were established to provide, analyze, and share data from multicenter series of patients, allowing an unprecedented exchange of knowledge.^13^–^20^

In this study, we describe a nationwide effort undertaken by the Italian Peritoneal Surface Malignancies Oncoteam—S.I.C.O. (Italian Society of Surgical Oncology), with the aim of investigating the short- and long-term outcomes of CRS/HIPEC in the treatment of synchronous PM from GC.

## Patients and Methods

### Questionnaire and Patient Selection

A questionnaire was created and sent to 11 high-volume and specialized Italian centers involved in CRS and HIPEC, from the Italian Peritoneal Surface Malignancies Oncoteam, in September 2019. The questionnaire included details about patients, perioperative chemotherapeutic regimens, pathologic reports, details of CRS and HIPEC, postoperative complications, and follow-up outcomes. Only patients with pathologically confirmed synchronous PM of GC (according to the 8th edition of the American Joint Committee on Cancer (AJCC) TNM classification^21^) and complete treatment, including gastrectomy with CRS and HIPEC, between 2005 and 2018, were included in the analysis. Exclusion criteria were any distant metastasis (except of the peritoneum) at the time of CRS and HIPEC. All patients provided informed consent for data recording in the registry and were treated according to multidisciplinary recommendations. Due to the retrospective nature of the anonymized data analysis, no Institutional Review Board approval was needed.

### Operative Strategy

According to the recommendations of the multidisciplinary team, patients underwent similar management with CRS and HIPEC in combination with neoadjuvant chemotherapy (NACT) whenever possible. After completion of NACT, tumor response was assessed using the new Response Evaluation Criteria in Solid Tumors (RECIST) guidelines version 1.1.^22^ In all centers, CRS and HIPEC were performed by a multidisciplinary team (surgeons, anesthesiologists, and operating room staff) specialized in peritoneal surgery and in the management of intraoperative chemotherapy. The extent of PM was assessed using the Peritoneal Cancer Index (PCI)^23^ immediately before CRS procedures, along with lavage peritoneal sampling; however, lavage cytology was not routinely performed in all patients prior to NACT and prior to CRS. Definitive CRS was performed in accordance with the techniques described by Sugarbaker 24, aiming at achieving complete cytoreduction. To reach this goal, patients underwent total or subtotal gastrectomy with D2 lymphadenectomy^25^ to remove the primary tumor, followed by peritonectomy procedures and visceral resections on demand in order to remove all visible peritoneal implants. After cytoreduction, and before HIPEC, all restorative anastomoses were completed and surgical radicality was determined according to the completeness of cytoreduction score (CCS).^26^ CCS-0 indicates no visible residual tumor and CCS-1 indicates residual tumor nodules ≤ 2.5 mm, while CCS-2 and CCS-3 indicate residual tumor nodules between 2.5 mm and 2.5 cm, and > 2.5 cm, respectively.^27^ HIPEC was performed using different protocols, depending on the center’s preferences, that differ in exposure technique, time, drugs, temperature. Postoperative mortality was defined as death within 90 days of surgery, while postoperative morbidity was recorded and scored according to the Clavien–Dindo classification system.^28^ Toxicity was assessed according to the National Cancer Institute Common Terminology Criteria for Adverse Events version 4.0 (NCI-CTCAE V4.0). After hospital discharge and complete recovery from surgery, patients received systemic chemotherapy possibly combined with biologic therapy according to their general status and the center’s protocols.

### Follow-Up

Patients were regularly followed-up after surgery, either in the surgical or oncological department, with blood tests (including tumor markers) and computed tomography every 3 months for the first 2 years, every 6 months from years 3–5, and yearly thereafter, or on demand, at any time, according to clinical status.

### Study Endpoints and Definition

The primary endpoints of this study were overall survival (OS), calculated from the date of CRS and HIPEC to the date of death by any cause or last contact, and recurrence-free survival (RFS), calculated from the day of CRS and HIPEC until the date of locoregional or distant recurrence. The secondary endpoints were analysis of morbimortality and prognostic factors for survival.

### Statistical Analysis

Descriptive statistics were reported as median (minimum and maximum values) or frequency (percentage). The estimated median follow-up time was obtained according to the reverse Kaplan–Meier method.^29^ OS and RFS analyses were performed using the Kaplan–Meier estimation method and compared using the log-rank test. All variables, which showed a *p*-value below 0.05 in univariate analysis, were included in the Cox regression model. Cox proportional hazards regression was used to calculate the hazard ratios (HRs) and 95% confidence intervals for the risk factors. A *p*-value < 0.05 was considered statistically significant. All statistical analyses were performed using the SPSS version 26.0 software package for Mac (IBM Corporation, Armonk, NY, USA).

## Results

### Patient and Treatment Characteristics

Of the 212 patients in the national multicentric database, 121 patients were excluded since, among them, 64 (30.2%) were submitted to prophylactic HIPEC, 31 patients (14.6%) were treated for metachronous peritoneal disease and 26 patients (12.3%) were submitted to laparoscopic HIPEC with palliative intent. A complete dataset of 91 patients fulfilling the selection criteria was available for this study. At preoperative work-up, a median PCI of 7 (range 2–34) was found. No data regarding the number of patients submitted to staging laparoscopy were available. In total, 60 (65.9%) patients received a median of 6 (1–14) cycles of preoperative chemotherapy using epirubicin, cisplatin, and fluorouracil or capecitabine (ECF/ECX) in the majority of patients. During NACT, 8 patients (8.8%) had grade 3 neutropenia and 12 (10.9%) patients had grade 3 anemia. These 20 patients received growth factor and only 5 patients needed NACT dose reductions. After NACT, restaging according to the RECIST criteria showed that 2 patients (3.3%) had complete response, 30 cases (50%) had partial response, and 28 cases (46.7%) presented with stable disease. Table 1 summarizes the clinicopathologic characteristics of selected patients. Peritoneal cytology results were positive for metastasis in 33 patients (36.3%), negative in 51 patients (56%), and non-diagnostic in 7 patients (7.7%). Interestingly, among 60 patients treated with NACT, only 3 patients (5%) experienced free peritoneal cancer (PC) cells.

**Table 1:** Patient demographic and clinical characteristics

Characteristics	Overall (*n* = 91)
Sex	
Female	45 (49.5)
Male	46 (50.5)
Age, years [median (range)]	58 (26–75)
ECOG performance status	
0	33 (36.3)
1	43 (47.2)
2	15 (16.5)
Location of tumor	
Upper third	17 (18.7)
Middle third	42 (46.1)
Lower third	29 (31.9)
Linitis plastica	3 (3.3)
Histological Lauren subtype	
Intestinal	27 (29.7)
Diffuse/mixed	64 (70.3)
Grading^a^	
G2	27 (29.7)
G3	64 (70.3)
Nodal status	
N negative	15 (16.4)
N positive	76 (83.6)
Lymphovascular invasion^a^	
No	22 (24.2)
Yes	69 (75.8)
Perineural invasion^a^	
No	40 (43.9)
Yes	51 (56.1)
Neoadjuvant chemotherapy	
No	31 (34.1)
Yes	60 (65.9)
Number of cycles [median (range)]	6 (1–14)

Data are expressed as *n* (%) unless otherwise specified

*ECOG* Eastern Cooperative Oncology Group

^a^Percentages are given according to the number of patients per line after the exclusion of patients with potential missing data

### Cytoreductive Surgery

At surgery, the median PCI was 6 (range 1–39). Forty-nine patients (53.8%) showed a median PCI of ≤ 6 and 42 patients (46.2%) showed a median PCI of > 7. The incidence and type of resection of additional organs to reach a CCS-0 status, as well as other operative details, are listed in Table 2.

**Table 2:** Surgical treatment and peritonectomy procedures in the overall patients

Characteristics	Overall [*n* = 91)
Surgical gastric procedure	
D2 partial gastrectomy	4 (4.4)
D2 total gastrectomy	87 (95.6)
Visceral resections^a^	
Splenectomy	49 (53.8)
Distal pancreatectomy	4 (4.4)
Small bowel resection	20 (21.9)
Appendectomy	33 (36.3)
Right colectomy	15 (16.5)
Left colectomy	4 (4.4)
Sigmoidectomy/proctectomy	9 (9.9)
Total colectomy	4 (4.4)
Hysteroannessiectomy	28 (30.8)
Cholecystectomy	66 (72.5)
Peritonectomies^a^	
Total parietal peritonectomy	28 (30.8)
Right upper quadrant	22 (24.2)
Left upper quadrant	23 (25.3)
Pelvic peritoneum	38 (42.8)
Mesenteric	18 (19.8)
PCI at CRS and HIPEC	
≤ 6	49 (53.8)
> 6	42 (46.2)
CCS	
CCS 0	73 (80.2)
CCS > 0	18 (19.8)
CCS 1	13 (14.3)
CCS 2	4 (4.4)
CCS 3	1 (1.1)
Surgical time, min [median (range)]	460 (120–755)
HIPEC duration, min [median (range)]	60 (30–90)
HIPEC technique	
Open	16 (17.6)
Close	75 (82.4)
HIPEC temperature, °C [median (range)]	42 (40–42)

Data are expressed as *n* (%) unless otherwise specified

*CCS* completeness of cytoreduction score, *°C* degrees Celsius, *PCI* Peritoneal Cancer Index, *HIPEC* hyperthermic intraperitoneal chemotherapy, *CRS* cytoreductive surgery

^a^Multiple answers were possible

### Heated Intraperitoneal Chemotherapy

The majority of centers performed a closed HIPEC technique (75 patients, 82.4%). The median temperature was 42 °C (range 40–42) and the mean duration of intra-abdominal chemotherapy was 60 min (range 30–90). Cisplatin, mitomycin C, and oxaliplatin were mostly used as HIPEC drugs. Two drugs (cisplatin and mitomycin C) were simultaneously administered in 36 patients (39.6%). Single-drug HIPEC with mitomycin C was administered in 11/55 patients (20%), cisplatin was administered in 20/55 patients (36.4%), and oxaliplatin was administered in 24/55 patients (43.6%). The median dose of cisplatin, mitomycin C, and oxaliplatin was 141 mg/m^2^ (range 77–350), 24.5 mg/m^2^ (range 10–54), and 360 mg/m^2^ (range 360–540), respectively.

### Postoperative Outcomes

After surgery, all patients were transferred to the intensive care unit for recovery until all vital signs were stabilized. HIPEC induced toxicity in 8 patients (8.8%): grade 1–2 acute kidney injury in two patients, grade 3 thrombopenia in two patients, and grade 3 leukopenia in four patients, promptly reversed by medical treatment. Clavien–Dindo classification grade 3b or higher occurred in 27 patients (29.7%), including six deaths (6.6% 90-day mortality); two of these patients developed acute myocardial infarction, one patient had a massive hemoperitoneum requiring an unsuccessful surgical intervention, and, in the remaining three cases, the cause of death was respiratory failure secondary to acute respiratory distress syndrome, septic shock in the context of anastomotic complication, and acute liver failure, respectively. After surgery, 71 patients (78%) completed systemic chemotherapy, while the remaining patients were not able to start the treatment due to postoperative complications that delayed discharge and recovery. Table 3 summarizes the main postoperative outcomes in the overall patients.

**Table 3:** Main postoperative outcomes in the overall patients

Postoperative outcomes	Overall (*n* = 91)
ICU hours [median (range)]	12 (0–240)
90-day mortality	6 (6.6)
Length of hospital stay, days [median (range)]	17 (7–93)
Postoperative medical complications^a^	
Respiratory	19 (20.9)
Cardiovascular	4 (4.4)
Renal	6 (6.6)
Urinary	4 (4.4)
Hepatic	1 (1.1)
Postoperative surgical complications^a^	
Anastomotic leakage	8 (8.8)
Hemorrhage	10 (11)
Intestinal obstruction/ileus	1 (1.1)
Pancreatic fistula	6 (6.6)
Small bowel perforations	3 (3.3)
Deep abscess	10 (11)
Wound dehiscence	3 (3.3)
Overall complications^a,b^	
1	5 (5.5)
2	10 (10.9)
3a	10 (10.9)
3b	15 (16.5)
4	6 (6.6)
5	6 (6.6)

Data are expressed as *n* (%) unless otherwise specified

*ICU* intensive care unit

^a^Multiple answers were possible.

^b^Overall complications are reported according to the Clavien–Dindo classification for grading complications

### Survival Analysis

**Fig. 1:**
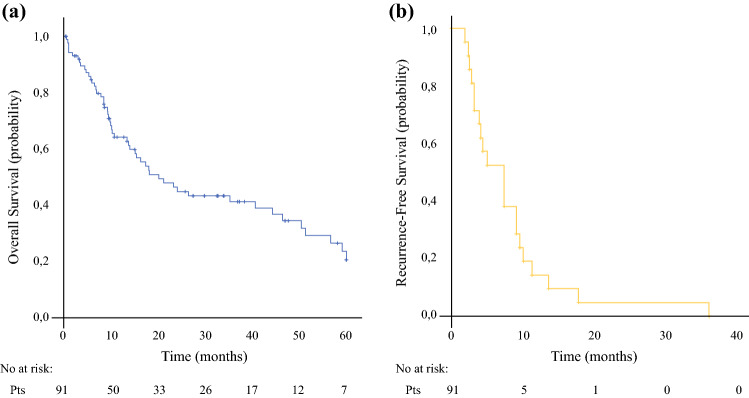
Kaplan–Meier survival curves of patients with peritoneal metastases of gastric carcinoma treated with cytoreductive surgery and hyperthermic intraperitoneal chemotherapy. **a** Overall survival; **b** recurrence-free survival. *Pts* patients

**Fig. 2:**
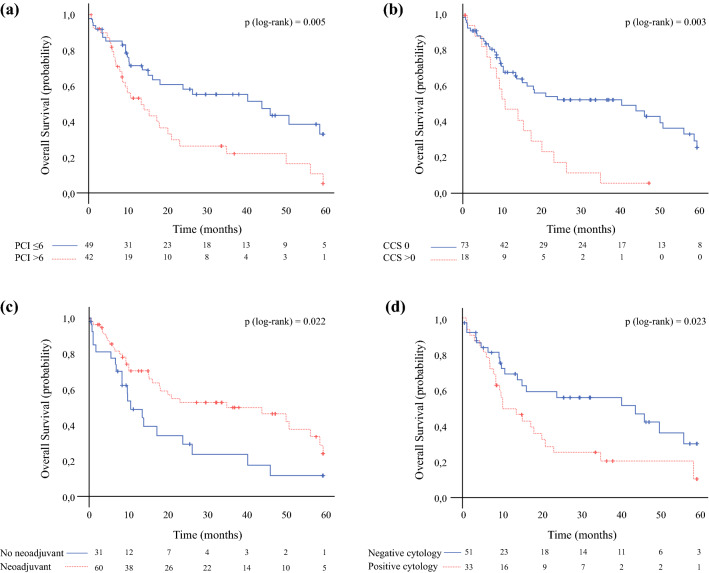
Overall survival stratified by **a** PCI ≤ 6 and PCI > 6; **b** CCS-0 and CCS > 0; **c** no neoadjuvant and neoadjuvant therapy; and **d** negative cytology and positive cytology. *PCI* Peritoneal Cancer Index, *CCS* completeness of cytoreduction score

The median follow-up was 47 months and no patients were lost to follow-up. The median OS time for the entire group of patients was 20.2 months (95% CI 11.8–28.5), with 1-, 3-, and 5-year OS rates of 62%, 44%, and 20.4%, respectively (Fig. 1a). The median RFS was 7.3 months (95% CI 4–10.6), with 1- and 3-year RFS rates of 14.3% and 4.8%, respectively (Fig. 1b). We ran survival analysis, grouping the PCI into two categories: PCI ≤ 6 (group 1, *n* = 49), and PCI from 7 to 39 (group 2, *n* = 42). In group 1, the median OS was 44.3 months (95% CI 16.4–72.1) and in group 2, the OS was 13.4 months (95% CI 6.2–20.5), with 5-year OS of 33.2% and 5.5%, respectively (*p* = 0.005) (Fig. 2a). The CCS-0 group showed better long-term survival compared with the incomplete resected group, reaching a high level of significance (*p* < 0.003). The median survival (months) was 40.7 (95% CI 11.7–69.7) for the CCS-0 group and 10.7 (95% CI 4.4–17) for the CCS > 0 group (Fig. 2b). The 5-year OS rate was higher for the CCS-0 group than the CCS > 0 group (25.9% vs. 5.9%). A significant difference was also observed in the survival rate according to NACT (untreated patients: 10.7 months, 95% CI 5.1–16.2; treated patients: 35.3 months, 95% CI 2.8–67.8; *p* = 0.022) (Fig. 2c). Finally, the median OS for patients with positive peritoneal cytology was worse than that of patients without free peritoneal metastatic cells (10.3 months [95% CI 3.9–17.6] vs. 44.3 months [95% CI 14.7–73.9]; *p* = 0.023) (Fig. 2d). We also investigated the differences in OS between patients according to other parameters (Table 4). The increase in mortality risk was nearly twofold in patients with PCI > 6 and CCS > 0 compared with those with PCI ≤ 6 and CCS-0. After adjusting for NACT, patients with PCI > 6 had a significantly increased risk of mortality compared with those with PCI ≤ 6. On the other hand, patients with CCS > 0 still had an increased risk but this was not statistically significant (Table 5). Further adjustments for peritoneal cytology and nodal status were made but had little effect on the HR.

**Table 4:** Univariate analysis of factors affecting survival

Variable category	*N*	Median overall survival (months)	Univariate analysis (*p *value)
Sex			
Male	47	21.2 (3.2–50.7)	0.542
Female	45	18.2 (9.4–26.7)	
Age, years			
< 60	49	18.2 (5.7–30.6)	0.877
≥ 60	39	20.2 (7.1–33.2)	
Lymph node metastasis			
Negative	15	60	**0.016**
Positive	76	13.7 (7.3–20)	
Lymphovascular invasion			
Negative	22	18.2 (0–37.5)	0.474
Positive	69	21.2 (13.7–28.6)	
Histology (Lauren)			
Intestinal	27	56.7 (3.3–110.1)	0.091
Diffuse	64	17.4 (10.9–23.8)	
Perineural invasion			
Negative	40	26.5 (1.7–51.3)	0.857
Positive	51	20.2 (13.4–27)	
Peritoneal Cancer Index			
≤ 6	49	44.3 (16.4–72.1)	**0.005**
> 6	42	13.4 (6.2–20.5)	
Cytoreduction			
CCS-0	73	40.7 (11.7–69.7)	**0.003**
CCS > 0	18	10.7 (4.4–17)	
Preoperative chemotherapy			
Yes	60	35.3 (2.8–67.8)	**0.022**
No	31	10.7 (5.1–16.2)	
Preoperative chemotherapy			
≤ 4 cycles	26	35.3 (5.8–64.7)	0.995
> 5 cycles	34	50.5 (2.7–98.3)	
Peritoneal cytology			
Negative	51	44.3 (14.7–73.9)	**0.023**
Positive	33	10.3 (3.9–17.6)	

Overall survival is illustrated as median (95% confidence interval)

*CCS* completeness of cytoreduction score

**Table 5:** Hazard ratios for risk factors of mortality.

Risk factor	HR (95% CI)	*p* value
Peritoneal Cancer Index		
≤ 6		
> 6	2.03 (1.11–3.7)	**0.02**
Cytoreduction		
CCS-0		
CCS > 0	1.66 (0.85–3.22)	0.135
Preoperative chemotherapy (NACT)		
Yes		
No	2.04 (1.14–3.64)	**0.017**

*HR* hazard ratio, *CI* confidence interval, *CCS* completeness of cytoreduction score, *NACT* neoadjuvant chemotherapy

## Discussion

The Italian Peritoneal Surface Malignancies Oncoteam network shows encouraging results, since, among a total of 91 enrolled patients, median OS was 20.2 months, with a 5-year OS rate of 20.4%. Several retrospective studies have identified predictive factors for patient selection regarding patients treated with CRS and HIPEC in the curative treatment of synchronous PM from GC.^10^,^11^,^18^,^30^ Nevertheless, the limited number of patients per study, as well as the consistent rate of incomplete or noncurative surgeries due to high peritoneal tumor burden, could affect the results.^3^,^7^,^11^,^20^,^31^–^36^

### Peritoneal Cancer Index

It is well-established that the lower the quantitative expression of tumor burden, expressed as PCI, the greater the chance to obtain complete CRS.^37^ However, if on the one hand the role of PCI as a prognostic factor is clear, on the other hand the cut-off for favorable prognosis is still debated.^3^,^11^,^20^,^30^,^38^ French and Japanese groups support a cut-off level of ≤ 6 or 7 according to their retrospective series,^11^,^39^ even if a cut-off of 12 does not seem unreasonable.^10^,^11^,^40^ In 81 patients with GC and PC from five French institutions treated with complete CRS and HIPEC, Chia et al.^37^ reported a median OS for patients with PCI < 7 of 26.4 months versus 10.9 months for patients with PCI ≥ 7. This same PCI cut-off also proved significantly prognostic for survival in the study by Yonemura^41^ (median OS of 33 months for PCI < 7 vs. 13 months for higher PCI scores), and, more recently, in a multicenter study of Spanish Group of Peritoneal Oncologic Surgery (patients with PCI ≤ 6 had a median OS of 26.1 months vs. 18.1 months for patients with PCI > 6).^8^ On the other hand, Coccolini et al.^40^ proposed a PCI score of 12 as the cut-off point for selecting patients with GC and PC for CRS and HIPEC. Survival was significantly better for PCI < 12, with a 3-year OS rate of 33% for PCI ≤ 6, 18% for PCI from 7 to 12, and 0% for PCI ≥ 13. Taking this evidence into consideration, in our cohort, patients with PCI  ≤  6 (53.8%) had a median OS of 44.3 months and a 5-year OS of 33.2%. Additionally, the mortality risk was doubled in patients with PCI > 6 compared with patients with PCI ≤ 6. Based on these results, we propose a PCI score of 6 as the cut-off point for selecting patients with PM from GC for CRS and HIPEC, excluding such treatment in patients with a higher PCI.

### Completeness of Cytoreduction Score

In their systematic review of 17 studies on the treatment outcomes of CRS and HIPEC for PM from GC, Chia et al.^32^ reported a median OS ranging from 6.6 to 15.8 months, with a 5-year survival rate of between 6% and 31%. Our favorable OS can be partly explained by the high quality of surgery since we obtained complete cytoreduction (CCS-0) in 80.2% of enrolled patients. Furthermore, all patients were treated at Italian Peritoneal Surface Malignancies Oncoteam centers, which include only recognized high-volume Italian centers with specific competence in peritoneal and gastric malignancies. Interestingly, our findings also confirm the crucial role of complete cytoreduction on survival outcomes. The CCS-0 group showed a significantly better long-term survival (median OS 40.7 months) compared with the incomplete resected groups (median OS 10.7 months). Similarly, in a series of 159 patients from 15 institutions that included patients with gastric PC treated with CRS and HIPEC, Glehen et al.^11^ reported a median OS of 15 months for CCS-0 and extremely low benefit for CCS ≥ 1 (median OS 6–8 months). In a recent large propensity score analysis of 277 patients by Glehen et al.,^13^ minimal residual disease (CCS-1) was associated with worse prognosis, resulting in a 5-year OS of 24.8% in CCS-0, compared with only 6.2% in the CCS-1 group. Accordingly, our survival analysis is in line with these findings. However, completeness of cytoreduction was found to be a prognostic factor in several other studies,^3^,^8^,^20^,^37^ suggesting CCS-0 as mandatory before a HIPEC procedure.

### Neoadjuvant Chemotherapy

The poor survival rate of patients who experienced incomplete or noncurative surgery due to a high PCI may be improved by perioperative chemotherapies. Accordingly, Valle et al.^42^ reported that CCS-0 can be realized in <30% of patients managed with upfront surgery, suggesting that patients with a PCI higher than the cut-off level at preoperative laparoscopy should be treated by NACT in order to reduce the tumor burden and accrue good prognosis after CRS and HIPEC. Currently, the Italian Research Group for Gastric Cancer (GIRCG) guidelines for the diagnosis and treatment of GC recommend neoadjuvant treatment for GC T ≥ 3 and/or with metastatic nodes on preoperative work-up.^43^ Nevertheless, there are still limited data about the effect of preoperative chemotherapy in patients with peritoneal metastases (PM) of GC. Our study was able to demonstrate a potentially beneficial effect of NACT on OS in patients eligible for CRS and HIPEC. Interestingly, among 60 patients treated with NACT, 51 (85%) presented with negative cytology and only 3 (5%) presented with free PC cells. However, 34.1% of patients were not treated with NACT and the reasons why were not stated in our registry, therefore remaining unclear. Potential reasons for this issue could be patients being operated before the introduction of NACT in the Italian guidelines. A significant difference was observed in the median OS according to NACT between both groups (untreated patients: 10.7 months; treated patients: 35.3 months). We also confirmed NACT favorable prognostic effect on OS in a Cox regression hazard model (HR 1.97, *p* < 0.049). Interestingly, the median OS in patients receiving four or fewer cycles of NACT was lower when compared with patients receiving more than five cycles (35.3 and 50.5 months, respectively), with no statistical significance. The number of NACT cycles actually represents an unresolved issue, since conflicting results have been published in the literature. In a randomized phase III study, Yang et al.^12^ reported improved OS for a cohort with more than six cycles, while Rau et al.^10^ demonstrated a potential negative effect of prolonged preoperative intravenous chemotherapy on OS. The differences could be explained on the basis of the heterogeneity of NACT drug protocols. We administered the protocol consisting of epirubicin, cisplatin, and fluorouracil or capecitabine (ECF/ECX) in the majority of patients. Preoperative treatment in the German group, instead, included a triple combination of oxaliplatin, leucovorin, and docetaxel, with taxane (FLOT).^44^ Obviously, there could be other occult factors influencing long-term survival in these patients, such as the differences from a molecular as well as biological point of view.

### Lymph Node Metastasis and Positive Cytology

Patients at risk of developing PM after curative resection for GC include those with nodal invasion and those with positive cytology.^45^–^47^ It is well-established that the impact of lymph node metastasis on OS, as well as tumor recurrence for GC patients, is extremely negative.^48^,^49^ Nevertheless, there is still limited evidence about the role of nodal involvement in patients with PM in GC treated with CRS and HIPEC. On this topic, Rau et al.^10^ reported a significantly improved median OS for N0 (26.7 months) rather than N+ (9.3 months) in a cohort of 58 patients treated with CRS and HIPEC. Accordingly, our data were able to demonstrate a negative effect of nodal involvement on survival outcomes. The median OS for patients with positive nodes was worse than that of patients without metastatic lymph nodes (13.7 months vs. 60 months; *p* = 0.016). It is likely the minor tumor burden of nodal-negative patients, from a systemic point of view, could lead to better response after CRS and HIPEC; however, further research into the genetic, molecular, and biological aspects of PM from GC may help identify tumor-specific markers associated with prognosis.

An important finding of our analysis is the significant difference between patients with positive peritoneal cytology at CRS (10.3 months) and patients without free peritoneal metastatic cells (44.3 months), resulting in positive cytology being a negative prognostic factor in Cox regression hazard models. However, in spite of this interesting evidence, the published data are scarce and it appears challenging to provide a robust explanation. It is well-recognized that patients with positive cytology have an 81% risk of peritoneal disease after curative surgery, as opposed to 45% for those with negative cytology.^50^ Nonetheless, to the best of our knowledge, no studies have been conducted investigating the role of circulating metastatic cells in patients with PM from GC. We highlight the favorable impact of negative cytology on peritoneal metastatic patients as a result of GC, postulating that these patients might have less malignant tumor biological characteristics and therefore benefit from CRS and HIPEC. Additionally, further studies exploring the application of new therapeutic tools, such as extensive intraoperative peritoneal lavage (EIPL),^51^ as well as bidirectional chemotherapy,^52^ are needed to help us better select which patients may be suitable for CRS and HIPEC. Interestingly, in the last century, attention has been focused on repeated intraperitoneal chemotherapy (RIPEC) using taxanes at normothermic conditions, mainly in the Eastern Countries.^53^ Even if the mechanism for shrinkage of peritoneal tumors has not been fully understood, repeated doses of intraperitoneal taxanes combined with systemic chemotherapy as neoadjuvant intraperitoneal and systemic chemotherapy (NIPS) resulted in an impressive improvement of survival outcomes (15.1–30.5 months) in connection with manageable toxicities.^54^–^56^ This tool may be a promising strategy for the management of peritoneal disease for GC and this contextual insight could be useful for developing hypotheses for further study.

### Limitations

One major limitation of our multicentric collaborative study is the heterogeneity of the selection criteria and management between institutions performing CRS and HIPEC. Additionally, the multicentric and retrospective nature of the data may affect some subjective results, such as intraoperative PCI and CCS. Nonetheless, this is one of the most important national registries, including only data from recognized high-volume Italian centers with specific competence in peritoneal and gastric malignancies, published to date.

## Conclusions

Although GC with PC still has poor prognosis, CRS and HIPEC after NACT, in referral centers, significantly improved survival in selected patients. Patients with a PCI score ≤ 6, complete cytoreduction, negative nodal involvements, and negative cytology experienced encouraging results, showing a clinically meaningful survival.
